# Genome-wide Cas9 binding specificity in *Saccharomyces cerevisiae*

**DOI:** 10.7717/peerj.9442

**Published:** 2020-07-29

**Authors:** Zachary J. Waldrip, Piroon Jenjaroenpun, Oktawia DeYoung, Intawat Nookaew, Sean D. Taverna, Kevin D. Raney, Alan J. Tackett

**Affiliations:** 1 Department of Biochemistry and Molecular Biology, University of Arkansas for Medical Sciences, Little Rock, AR, United States of America; 2 Department of Biomedical Informatics, University of Arkansas for Medical Sciences, Little Rock, AR, United States of America; 3 Department of Pharmacology and Molecular Sciences, Johns Hopkins School of Medicine, Baltimore, MD, United States of America

**Keywords:** CRISPR, ChIP-seq, Chromatin Immunoprecipitation, Cas9, Off-target binding

## Abstract

The CRISPR system has become heavily utilized in biomedical research as a tool for genomic editing as well as for site-specific chromosomal localization of specific proteins. For example, we developed a CRISPR-based methodology for enriching a specific genomic locus of interest for proteomic analysis in *Saccharomyces cerevisiae*, which utilized a guide RNA-targeted, catalytically dead Cas9 (dCas9) as an affinity reagent. To more comprehensively evaluate the genomic specificity of using dCas9 as a site-specific tool for chromosomal studies, we performed dCas9-mediated locus enrichment followed by next-generation sequencing on a genome-wide scale. As a test locus, we used the *ARS305* origin of replication on chromosome III in *S. cerevisiae*. We found that enrichment of this site is highly specific, with virtually no off-target enrichment of unique genomic sequences. The high specificity of genomic localization and enrichment suggests that dCas9-mediated technologies have promising potential for site-specific chromosomal studies in organisms with relatively small genomes such as yeasts.

## Introduction

Elements of the CRISPR (Clustered Regularly Interspaced Palindromic Repeats) bacterial immune system from *Streptococcus pyogenes* have been transformed into a popular and efficient genome editing system (DiCarlo et al., 2013; Doudna & Charpentier, 2014; Jinek et al., 2012; Mali et al., 2013; Wiedenheft, Sternberg & Doudna, 2012). By expressing the Cas9 protein and a short guide RNA molecule (gRNA), it is possible to induce targeted double-stranded breaks in eukaryotic cells. This can result in indels (insertions/deletions) at the site of the induced break via the non-homologous end joining repair pathway to create random genetic mutations or can be exploited to insert precise sequences into the genome via the homologous recombination repair pathway (Doudna & Charpentier, 2014; Hsu et al., 2013; Mali et al., 2013). In addition to this, a catalytically inactive version of the Cas9 protein otherwise referred to as dead Cas9 (dCas9), has been used for many applications outside the field of genome editing. This version of the Cas9 protein contains single amino acid mutations within both the HNH and RuvC-like nuclease domains (Asp^10^ to Ala and His^840^ to Ala) (Qi et al., 2013). Cas9 binds a target DNA sequence in part through association with a guide RNA (gRNA). This gRNA typically contains a 20 bp sequence complementary to the genomic target and confers the majority of the target specificity. The Cas9 protein also recognizes an adjacent three-nucleotide sequence called the protospacer adjacent motif, or PAM, immediately 3′ to the 20 bp matching sequence.

Catalytically inactive Cas9 has been employed for a number of creative techniques for site-specific genome manipulation. One example of an alternative use of the CRISPR system includes gene silencing (CRISPRi(nterference)) where dCas9/gRNA is used to silence gene expression by acting as a physical barrier to transcription (Esvelt et al., 2013; Gilbert et al., 2013; Qi et al., 2013). Another example is genome regulation, which is accomplished by fusing an effector domain to dCas9 and targeting it to a genomic site of interest. This effectively turns dCas9 into a site-specific transcriptional activator or repressor (Cheng et al., 2013). A technique developed in our laboratory, which we termed CRISPR-ChAP-MS (CRISPR-Chromatin Affinity Purification-Mass Spectrometry), uses an affinity tagged dCas9 as a handle for isolating a targeted genomic locus for proteomic analysis (Waldrip et al., 2014). This particular technique was developed to isolate a single-target unit of chromatin (about 500 bp sections) from a cell lysate for subsequent protein identification by mass spectrometry. This gives the investigator insight into the regulatory protein complexes as well as histone post-translational modifications (PTMs) that may be driving the regulation of a target chromatin element (Byrum et al., 2012; Byrum, Taverna & Tackett, 2013; Fujita & Fujii, 2016; Waldrip et al., 2014).

The efficacy of all these techniques relies on the specificity of the dCas9/gRNA complex; that is, a high ratio of target-to-off target DNA binding. If the dCas9/gRNA complex is bound tightly to any secondary sites with similar sequences, this could cause off-target effects such as unaccounted gene disruption, transcriptional activation/repression, or chromatin purification (Cheng et al., 2013; Esvelt et al., 2013; Qi et al., 2013; Waldrip et al., 2014). This is the potential drawback of the CRISPR system (Fu et al., 2014; Koo, Lee & Kim, 2015; Kuscu et al., 2014; Wu et al., 2014). In an experiment designed to isolate chromatin bound by the dCas9/gRNA complex, every tagged Cas9 molecule in a lysate is a target of enrichment. Inevitably, background chromatin enrichment will occur. If this background is much lower than the target chromatin, it can be managed using proper controls. However, if there are off-target sites with high sequence complementarity to the gRNA sequence, they may be enriched at relatively high levels between those of the target and the background, leading to convolution of the data during detection and/or analysis. Because experiments using a catalytically inactive Cas9 can be more susceptible to the negative influence of off-target sites than experiments using catalytically active Cas9 (Wu et al., 2014), understanding the propensity of Cas9 to bind off-target sites relative to its target site within the context of a particular application is valuable.

To gauge global enrichment and address the question of whether off-target sites were significantly enriched using the CRISPR-ChAP method, we employed ChIP-seq for genome-wide evaluation of site-specific localization of dCas9/gRNA. We hypothesized that enrichment of a target locus using the CRISPR-ChAP method would be highly specific, with very few off-target sites of enrichment given the small size of the yeast genome and the uniqueness of our target sequence. Nonetheless, off-target interactions of Cas9 are well-documented and arguably the most important issue with the CRISPR system. So, we sought to evaluate this in a comprehensive manner with sequencing. ChIP-sequencing is one of the most thorough methods of identifying changes in genome-wide binding of chromatin binding proteins and is widely utilized in the CRISPR field (Kuscu et al., 2014; O’Geen et al., 2015; Qin et al., 2016; Wienert et al., 2019). We also sought to capitalize on the constant progress in the field of CRISPR biology by integrating some of the newer technology into our technique and making some adjustments to our methodology, including modifying the dCas9 expression level. There has also been a lot of effort put toward modifying the Cas9 protein itself for the purpose of more specific and efficient genome editing. We decided to compare one of these alternative Cas9 variants called enhanced Cas9 (1.1) with the traditionally used wild-type Cas9 to see if it could offer an improvement in chromatin enrichment by lowering off-target enrichment. This mutant version of Cas9 contains two lysine to alanine mutations and one arginine to alanine mutation at residues that interact with the negatively charged DNA phosphate backbone. By neutralizing these positively charged residues, binding stability becomes more dependent upon correct gRNA:DNA base pairing resulting in a reduction in genome-wide off-target cleavage for the catalytically active version (Slaymaker et al., 2016).

In short, our experimental design is as follows. Yeast lysates from strains expressing Protein A (PrA)-tagged dCas9 and a gRNA targeting the *ARS305* origin on chromosome III were used for affinity purification experiments to enrich dCas9/gRNA-bound chromatin. Before elution of the captured targets, the affinity resin (IgG-coated magnetic beads) was washed stringently with a urea-containing solution. Eluted DNA was purified from total chromatin, ligated to Illumina indexes, and prepared for NextGen sequencing. Analysis of the sequencing data revealed virtually no consistent off-target enrichment other than at the highly repetitive rDNA loci and high-copy expression plasmid, giving important insight into the relative efficiency of target chromatin enrichment by dCas9 from a global genomic viewpoint. It also lent insight into how to address potential pitfalls of methodologies utilizing plasmid-based expression of catalytically inactive Cas9

## Materials & Methods

### Chromatin Affinity Purification (ChAP)

*Saccharomyces cerevisiae* cultures (see *Construction of plasmids* for strain information) with pPrA-dCas9 + gRNA-ARS305 were grown to mid-log phase in synthetic yeast media (minus tryptophan) with 2% w/v raffinose and subjected to 1% formaldehyde cross-linking for 5 min. The reaction was quenched with 125 mM glycine for 5 min. Cells were collected by centrifugation, rinsed, snap frozen, and lysed under cryogenic conditions in a bead mill, as done previously (Byrum et al., 2012; Byrum, Taverna & Tackett, 2013; Waldrip et al., 2014). Lysate from 1.5 g of cells (∼5 × 10^10^ cells) was re-suspended at 4 °C in purification buffer (50 mM HEPES-KOH pH 7.5, 1 mM EDTA, 0.1% w/v deoxycholic acid, 1% v/v Triton X-100, 140 mM NaCl, 1× Sigma fungal protease inhibitor cocktail) at 5 mL/gram cell lysate. Re-suspended cell lysate was split into three 0.5 g (2.5 ml) samples and subjected to sonication for two 15-minute cycles (30s on/off) in a Bioruptor water bath sonicator to shear chromatin to ∼0.5 kb in size on average (∼0.2–1 kb). PrA-tagged dCas9/gRNA complex and associated chromatin were affinity purified on 5 mg of M270 Dynabeads conjugated to rabbit IgG (MP Biomedicals #55944) per gram of cells. IgG-coated beads were incubated with lysate for 3 h at 4 °C with gentle rotation. Beads were collected with magnets and washed using an updated version of the original wash protocol as follows: once in purification buffer, once with purification buffer with 1 M urea/1 M NaCl, once in purification buffer with 4 M urea/250 mM LiCl, once in purification buffer with 250 mM LiCl, and once more in purification buffer. All washes were done for 5 min with agitation except the first and last wash. DNA was eluted from the beads in 100 µl of 25 mM Tris pH 8, 2 mM EDTA, 1% SDS by heating for 20 min at 95 °C, followed by incubation at 65 °C overnight (∼16 h). The eluate was treated with 20 µg RNase A for 30 min, then 20 µg proteinase K for 30 min at 37 °C. The DNA was subsequently purified on silica columns using a Promega SV Gel and PCR cleanup kit. These samples were then used to generate 24 pooled paired-end indexed DNA libraries with the TruSeq ChIP Library Preparation Kit from Illumina following the instructions for NGS sample preparation with the Illumina TruSeq ChIP Sample Preparation Guide available from Illumina’s website at the following address: https://support.illumina.com/downloads/truseq_chip_sample_preparation_guide_15023092.html. Samples were sequenced on the Illumina NextSeq platform.

### Real-time PCR

Real-time PCR was performed using the ChAP DNA purified with a Promega PCR purification kit. Each PCR reaction was performed in technical triplicate using SYBR green detection with a Bio-rad CFX96 Touch Real-time PCR detection system. Data was analyzed using the ΔΔ*C*_*t*_ method to determine enrichment of the target locus relative to a sequence within the *ACT1* locus. Primers for qPCR are as follows: ARS305-1 target set 5′CTCTTCCTCTTCCTCGAAAGTC and 5′AGGTTCAGTGTCCCAATGAG, ARS305-2 target set 5′ATTGAGGCCACAGCAAGA and 5′TAAATCACACCGGACAGTACAT, ACT1 target set 5′TCAAATCTCTACCGGCCAAATC and 5′GATTCCGGTGATGGTGTTACTC.

### Construction of plasmids and ChAP control strain

*S. cerevisiae* strains (W303 matA *bar1* Δ, origin—established cell line from Dr. Brian Chait’s laboratory at The Rockefeller University) were created by transformation with a variation of the plasmid pPrA-dCas9 + gRNA-ARS305 with the *GAL1* promoter and either dCas9 or enhanced dCas9 (1.1) (edCas9 (1.1)). These plasmids were assembled from PCR fragments using the In-Fusion method (Clontech) followed by mutagenesis to change the 18 bp guide RNA target sequence (Fu et al., 2014). The ARS305-1 target sequence is GTTGGTAGCACTTTGATG, and the ARS305-2 target sequence is CCAGTTTCATGTACTGTC (the target site used for the sequencing study). Enhanced dCas9 (1.1) was generated by site-directed mutagenesis in the same manner as the guide RNAs.

The ChAP control strain was generated by homologous recombination to generate a genomic deletion of the *ARS305* locus. The *ARS305* consensus sequence was replaced with a *Kluyveromyces lactis URA3* gene + promoter. Selection for knockout cells was done on media lacking uracil. Cells were screened by PCR to verify *URA3* replacement at the correct genomic location. This strain was transformed with the same dCas9 + gRNA plasmid as the matched experimental strain.

For detailed contextual information regarding the *ARS305* locus, gRNA target sequences, and PCR primers see Fig. S1.

### ChAP-seq data analysis

Raw reads obtained from the ChAP libraries were first examined for quality using the FastQC software (version 0.11.2) from http://www.bioinformatics.babraham.ac.uk/projects/fastqc/. Then the possible remaining adaptors and raw reads were quality trimmed using Trimmomatic (Bolger, Lohse & Usadel, 2014) with the default setting to obtain high quality reads. The high-quality reads were mapped to the reference genome (*Saccharomyces cerevisiae* S288C, GCF_000146045.2), retrieved from the NCBI database along with the corresponding annotation files using BWA-MEM (Li & Durbin, 2010) followed by Stampy aligner (Lunter & Goodson, 2011) to improve mapping accuracy. Subsequently, duplicated reads were removed from alignments using MarkDuplicates (version 2.9.2) from Picards tools version 2.9.2 (https://broadinstitute.github.io/picard/command-line-overview.html#MarkDuplicates). The aligned reads were further filtered by removing those with an alignment quality score of less than 30 using Samtools (version 1.5) (Li et al., 2009). Data is available at NCBI Bioproject/SRA, accession number PRJNA574983.

Enriched peak regions of the genome were identified by comparing the ChAP samples to WCE samples using the Model-based Analysis for ChIP-Seq (MACS) software version 2.0 (Zhang et al., 2008) with the default setting. Bedtools were employed to identify all possible overlapping peaks, which were then collapsed into a single set of unique genomic intervals (a consensus peak set for all 3 replicates of each sample type). Consensus peak sets with overlapping sequences shorter than 200 nucleotides were excluded from the analysis. The identified peak sets and alignment (BAM) files were used as input for differential binding analysis using DiffBind (Ross-Innes et al., 2012), which utilizes DESeq2 for performing read normalization and differential analysis.

To investigate off-target sites, we used the Cas-OFFinder program (Bae, Park & Kim, 2014) with the seed and the 5′-NRG- 3′ PAM sequence to predict on- and off-target sites from the yeast reference genome as well as the dCas9/gRNA expressing plasmid. Following a previous study (Kuscu et al., 2014), we used a maximum number of mismatches of up to 9 bp for off-target analysis.

## Results

### Chromatin Affinity Purification (ChAP) Optimization and Enrichment of *ARS305*

**Figure 1:**
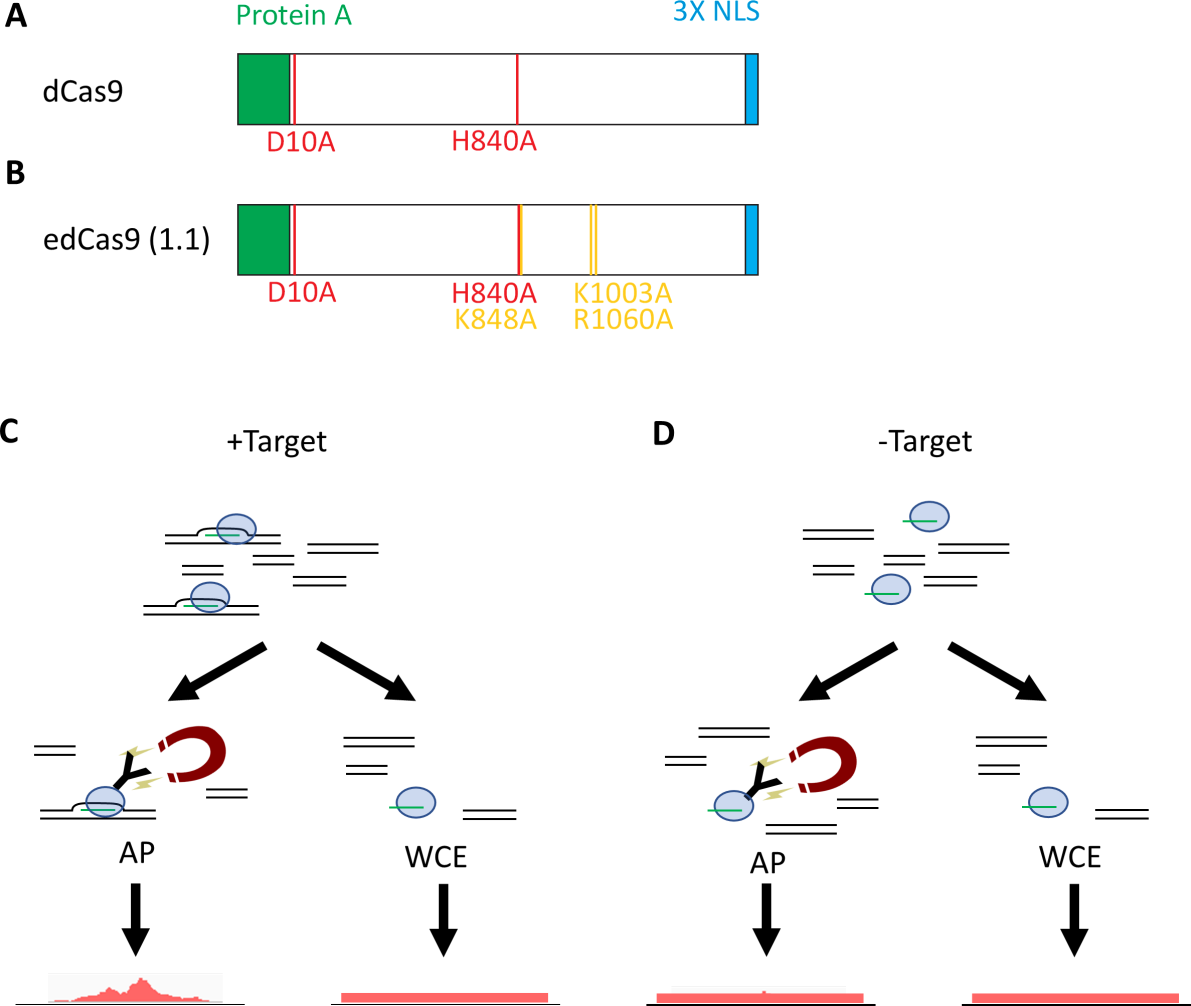
CRISPR-ChAP-sequencing experimental outline. Organization of the Cas9 constructs and the amino acid differences between the wild-type (WT) dCas9 (A) and edCas9 (1.1) (B). (C) and (D) show a schematic of the ChAP-seq approach. Cells containing the *ARS305* cassette (+Target) (C) and *URA3* cassette (−Target) (D) are transfected with plasmids encoding the dCas9 or edCas9 (1.1) nuclease and gRNA. Chromatin is purified using IgG-coated magnetic beads and both affinity-purified (AP) and whole cell extract (WCE) samples are sequenced and analyzed. Three biological replicates of each experiment are performed.

The goal of this work was to use next-generation sequencing to evaluate the specificity of dCas9 and a published mutant version of dCas9 under the conditions used for CRISPR-ChAP (Waldrip et al., 2014). For our studies, we enriched a target site near an early-firing origin of replication on chromosome III, *ARS305* (Dershowitz et al., 2007). We used an 18 bp truncated gRNA (tru-gRNA) with the guide sequence 5′CCAGTTTCATGTACTGTC paired with a catalytically inactive Cas9 protein containing the D10A and H840A mutations within the nuclease domains (Fig. 1A) (Fu et al., 2014; Jinek et al., 2012). We also wanted to compare the peak enrichment profile of an otherwise wild-type dCas9 and a mutant dCas9, which is designed to be more dependent upon the gRNA-target hybridization for efficient DNA binding. This enhanced Cas9, referred to as eCas9 (1.1), was shown to result in fewer off-target mutations when tested in the context of genome editing (Slaymaker et al., 2016). We were interested to see if this benefit also applied to Cas9 binding and enrichment, so we created a catalytically inactive version of this mutant, which we refer to as edCas9 (1.1) (Fig. 1B). For a control, a strain was designed in which the gRNA target site was removed from the genome and replaced with a gene cassette containing *URA3* and its promoter. This allowed for the expression of both the dCas9 protein and the gRNA in both the experimental and control strains, hypothetically preserving any off-target sites or other chromatin specifically interacting with the gRNA or dCas9/gRNA complex. A depiction of the experiment is given in Fig. 1C (experimental strain) and Fig. 1D (control strain).

**Figure 2:**
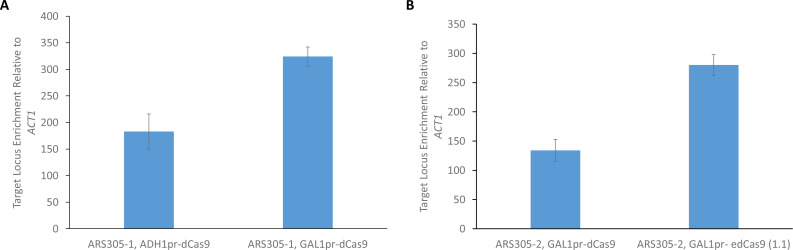
Enrichment of *ARS305* chromatin by ChAP. (A) Relative enrichment of *ARS305* chromatin was compared from cells expressing the ARS305-1 gRNA and dCas9 under control of either the *ADH1* promoter or the *GAL1* promoter (raffinose induction). (B) Relative enrichment of *ARS305* chromatin with dCas9/gRNA or edCas9 (1.1)/gRNA from samples used for ChAP-sequencing. This was conducted using plasmids expressing the ARS305-2 gRNA and dCas9 under control of the *GAL1* promoter. For both panels, qPCR was used to determine enrichment at the *ARS305* target site relative to a site within the *ACT1* locus with results from three biological replicates with indicated standard error.

Two target sites were used in this study. A target sequence referred to as ARS305-1 was used during most of the initial work on the methodology, and another site referred to as ARS305-2 was ultimately used for the sequencing experiment. These sites flank and are within 200 bp of the annotated *ARS305* origin sequence (Fig. S1). Initially, we compared enrichment with dCas9 expressed from either the *ADH1* promoter used in the previous version of our protocol or the more modular *GAL1* promoter with attenuated expression (*GAL1* promoter with 2% raffinose induction rather than galactose) (Fig. 2A). Expression of dCas9 with the *GAL1* promoter resulted in approximately twice the level of relative enrichment compared to the *ADH1* promoter. Given the higher level of relative enrichment observed when dCas9 expression was under the regulation of a raffinose-induced *GAL1* promoter, we proceeded with this promoter and growth condition in subsequent experiments.

We used these optimized ChAP conditions for enrichment of *ARS305* chromatin from the following four strains: dCas9/gRNA in wild-type yeast, dCas9/gRNA in yeast with genomic deletion of *ARS305*, edCas9 (1.1)/gRNA in wild-type yeast, and edCas9 (1.1)/gRNA in yeast with genomic deletion of *ARS305*. Cultures were grown to mid-log phase and cross-linked in formaldehyde. *ARS305* chromatin was enriched on IgG-coated magnetic Dynabeads in a strain expressing the ARS305-2 targeting gRNA (see methods for details). Enriched DNA was isolated for qPCR and next-generation sequencing. Relative enrichment was measured by qPCR (ARS305-2 PCR primer set), which showed 134-fold enrichment of *ARS305* relative to a non-target site within the *ACT1* locus from the strain expressing dCas9 and 280-fold from the strain expressing edCas9 (1.1) (Fig. 2B).

**Figure 3:**
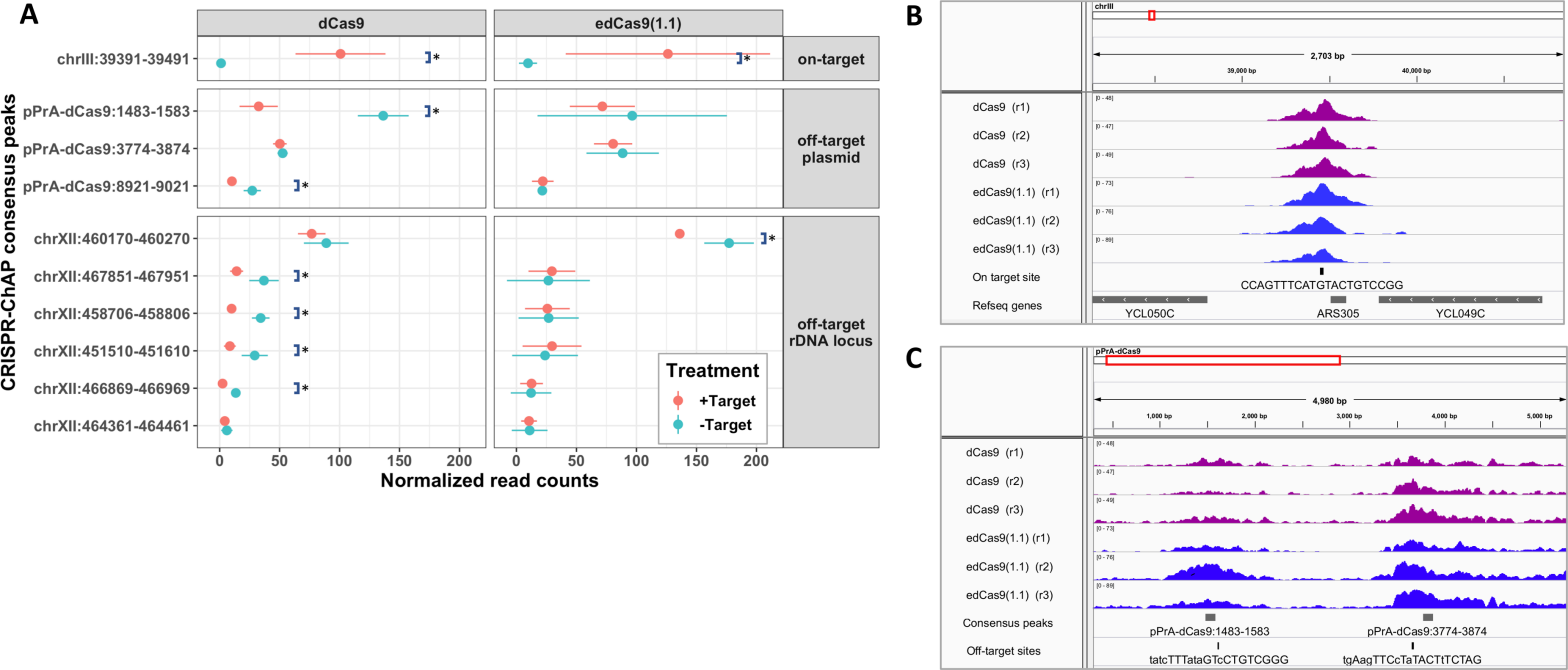
Specificity of dCas9/gRNA and edCas9 (1.1)/gRNA enrichment of *ARS305* chromatin. (A) The dot plot represents a comparison of the mean of normalized reads with standard deviation (*n* = 3) between + and − Target of dCas9/gRNA and edCas9/gRNA experiments calculated using DiffBind. The consensus peaks were categorized into either on-target or off-target based on general location (sequences inside the plasmid or the ribosomal DNA locus). Asterisks represent significant differences where *p* < 0.05. +Target indicates a wild-type *ARS305* locus, while −Target indicates genomic replacement of *ARS305* with *URA3*. ChAP-sequencing data for dCas9 and edCas9 (1.1) binding is represented visually at the on-target site at *ARS305* on chromosome III (B) and at potential off-target sites in the plasmid (C).

DNA was then indexed for sequencing using the Illumina® TruSeq ChIP Library Preparation Kit. Each sample was indexed using one of 24 Illumina oligonucleotide adapters, and all 24 samples were pooled and single-end sequenced on a single flow cell. Genome-wide binding sites were identified using the MACS peak caller using whole-cell extract (WCE) as background. To reduce the occurrence of false positive peaks, we selected genomic intervals which share common nucleotides of significantly enriched peaks among all 3 replicates (referred to as a ‘consensus peak’) (see Methods). Only 10 consensus peaks were identified by ChAP-sequencing (Fig. 3A). One of these consensus peaks was observed at the intended target site near *ARS305* (i.e., the on-target site, chrIII:39391-39491) (Figs. 3A and 3B). The other 9 sites were further evaluated as potential off-target sites (Figs. 3A and 3C).

To compare the differential enrichment of these consensus peak sets, DiffBind was used to identify significant differential binding of dCas9/gRNA and edCas9 (1.1)/gRNA in +Target (wild-type yeast) over −Target (yeast with *ARS305* replaced by *URA3*) experiments. As expected, the target site constitutes the most highly bound location (height of the ChAP-seq peak) for both dCas9 and edCas9 (1.1) (Figs. 3A and 3B and Table 1). The target site (+Target) was significantly enriched approximately 88.6 and 13.1 times higher than the target depleted site (−Target) in dCas9 and edCas9 (1.1), respectively (Table 1). Comparison of dCas9 and edCas9 (1.1) in the +Target samples shows little difference in the binding affinity of these two versions of dCas9 and did not reach statistical significance. Interestingly, six of the nine potential off-target sites from the dCas9 samples were enriched in the −Target samples compared to the +Target. In fact, one site located on the plasmid (pPrA-dCas9:1523-1573) was enriched in the −Target samples approximately 4-fold higher than in the +Target for the samples containing dCas9 (Figs. 3A and 3C and Table 1). This was the case for only one of these nine sites for the edCas9 (1.1) samples.

**Table 1:** Highly enriched sequences by CRISPR-ChAP sequencing.

**Condition**	**On/off-target location**	**Annotation**	**Mean of log2 normalized read counts**		**Fold**	***p* value**	**FDR**
			**+Target**	**−Target**			
dCas9 +Target vs. −Target	chrIII:39416-39466	On-target	6.66	0.19	88.6	9.9E−24	9.9E−23
	pPrA-dCas9:1523-1573	Off-target	5.02	7.09	4.2	2.4E−08	1.2E−07
edCas9 (1.1) +Target vs. −Target	chrIII:39416-39466	On-target	6.98	3.27	13.1	2.7E−05	2.7E−04

### Identification off-target binding sites for dCas9 and edCas9 (1.1)

**Figure 4:**
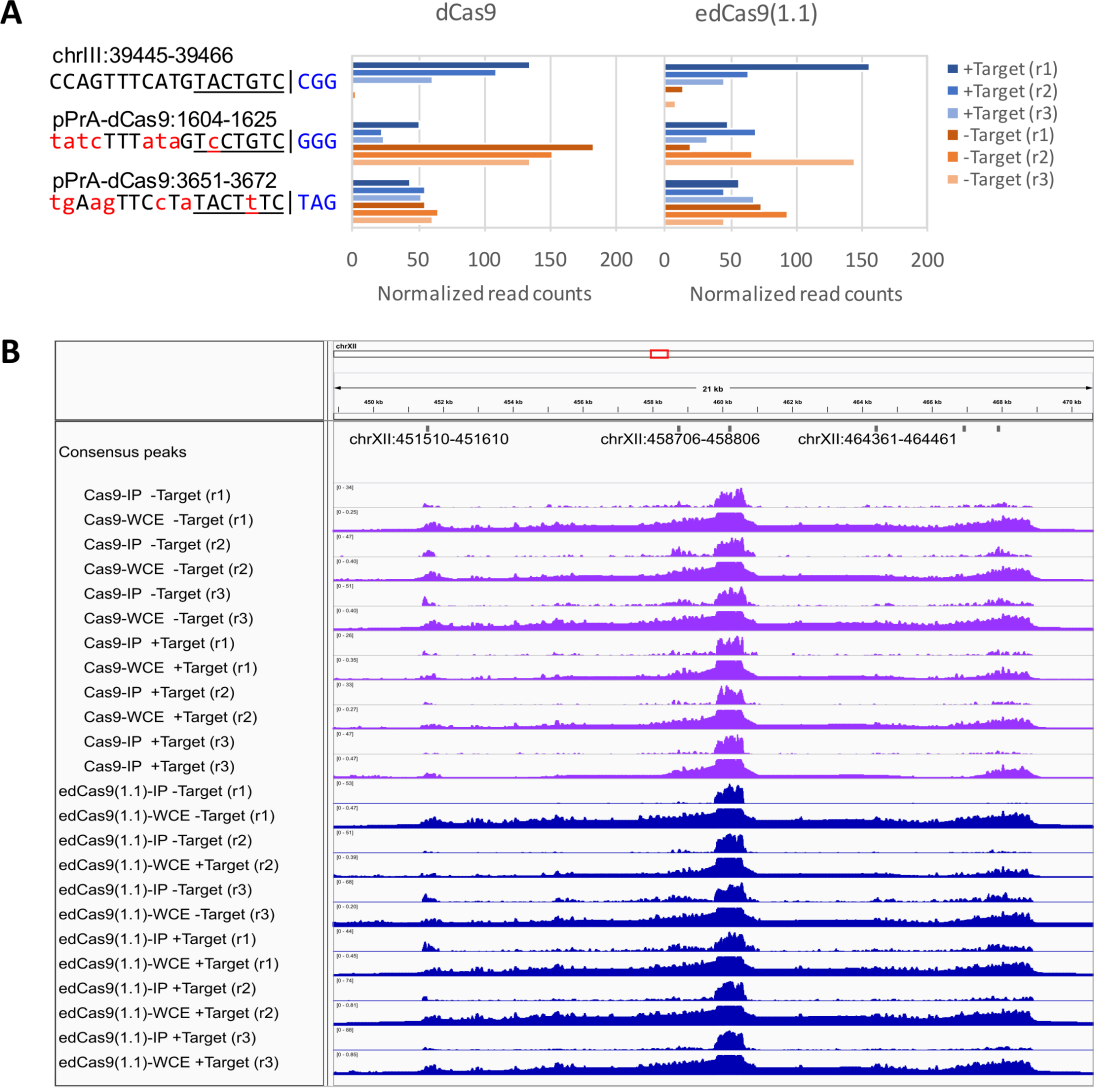
Analysis of consensus peaks at off-target sites. (A) The *ARS305* target site and plasmid sequences enriched by dCas9:gRNA are shown. This is a list of sequences within 150-bp from the center of the consensus peaks that contain at most 1 mismatch in the PAM-proximal 10-bp (7-bp seed sequence + 3-bp PAM). Bar graphs on the right present normalized read counts to the target site for each experimental sample listed on the far right. Mismatched bases are shown in red. The PAM sequence is shown in blue. (B) CRISPR-ChAP profiles of WT dCas9, edCas9(1.1), and WCE-seq at the rDNA loci on chromosome XII are illustrated.

Next, we explored the possibility that the other identified consensus peaks shared sequence identity with the target sequence using the Cas-OFFinder program (Bae, Park & Kim, 2014). We considered mismatches of up to 9 bp for off-target analysis and identified 1 on-target and 47,965 potential off-target sequences by this criterion. Previous studies have reported that the CRISPR-Cas9 system has far less tolerance to mismatches in the PAM-proximal compared to the PAM-distal region (Hsu et al., 2013; Kuscu et al., 2014). Therefore, we filtered out predicted off-target sequences that contain more than 1 mismatch in the PAM-proximal 10 bp (7-bp seed sequence + 3-bp PAM). Finally, we identified 1 on-target and 1,416 predicted off-target sequences. These predicted on- and off-target sequences were used for supporting ChAP-seq signals. There were three predicted sequences located within 150 bp of the consensus peaks (see Methods; ‘Off-target prediction’). This is shown in Fig. 4A with the ChAP-seq signal of each individual sample. One of these three sequences is the target site (chrIII: 39445-39466). The other two predicted off-target sequences are on the plasmid. The plasmid used in this study contains a multicopy 2µorigin, meaning there are multiple copies of the plasmid per cell. If the Cas9/gRNA complex has a slight affinity for any sequence within the plasmid, this could result in a greatly amplified signal above the background sequences. There are no predicted off-target sequences in the vicinity of the other seven consensus peaks. Six of these peaks fall among the highly repetitive rDNA loci near the centromere of chromosome XII (Fig. 4B). The seventh is on the multicopy plasmid.

## Discussion

One of the most important goals of this study was to determine what, if any, off-target sites are consistently occupied by dCas9. Most CRISPR studies find some amount of off-target binding and have found this can vary quite a bit depending on a number of factors including genome size, Cas9 expression level, and gRNA target sequence which makes a direct comparison with other prominent CRISPR ChIP-seq studies difficult (Kuscu et al., 2014; O’Geen et al., 2015; Wu et al., 2014). These types of CRISPR ChIP-seq studies using catalytically inactive Cas9 have been conducted almost exclusively in mammalian models with much larger genomes. This means that any given gRNA sequence has a much higher likelihood of aligning closely to another genomic sequence, therefore increasing the probability of off-target recognition by the gRNA. For example, the human genome is approximately 3.1 billion base pairs, whereas the *S. cerevisiae* genome is approximately 12 million (a 250-fold difference). One study observed anywhere from tens to more than 1,000 off-target binding sites depending on the chosen gRNA sequence in human HEK293T cells (Kuscu et al., 2014). Put into context, our finding of a lack of off-target enrichment at single-copy genomic sequences in the relatively small yeast genome is not all that surprising. In this study, we found enrichment of the *ARS305* locus with our gRNA sequence to be incredibly specific with respect to the genome. However, the exceptions to this include both highly repetitive sequences within the rDNA locus and the multiple-copy expression plasmid. This is not too surprising given that highly enriched regions such as those near centromeres, telomeres, and other repeated sequences are often considered artefacts in similar studies due to the highly repetitive nature of these sequences (Fu et al., 2014; Jenjaroenpun et al., 2018; Kuscu et al., 2014; Qin et al., 2016). Regarding the enriched sites within the plasmid, location pPrA-dCas9:1483-1583 maps to the bacterial origin of replication, location pPrA-dCas9: 3774-3874 maps to the 2µreplication origin, and pPrA-dCas9:8921-9021 maps to the dCas9 open reading frame. In addition to multiple copies of the plasmid being a probable contributing factor in the enrichment of these sequences, these sites in particular could possibly be more vulnerable to enrichment due to an open chromatin conformation given that replication origins are often relatively devoid of nucleosomes for efficient access (Eaton et al., 2010; Lipford & Bell, 2001; Tsai et al., 2014). However, this is only a speculative explanation. Overall, our results are likely due to a combination of several factors including stringent ChAP conditions, optimized expression of Cas9, and the relatively small size of the yeast genome.

In our study, we found that optimizing the level of dCas9 is an important factor to consider and test when designing a CRISPR experiment, as already demonstrated by other groups (Hsu et al., 2013; Pattanayak et al., 2013; Wu et al., 2014). We evaluated this by comparing DNA enrichment by qPCR from samples in which dCas9 expression was regulated by either the *ADH1* promoter (grown with glucose) or the *GAL1* promoter in the presence of raffinose. Generally, what we and others have found is that if levels of Cas9 protein are too high, this can result in unwanted binding and/or editing. Lower levels can give higher specificity, albeit at a possible cost in efficiency.

While there seems to be little difference between dCas9 and edCas9 (1.1) total target enrichment under the experimental conditions that we have tested, the dCas9 in general seems to have a higher affinity for the plasmid and rDNA sites in the absence of the intended target site (−Target) (6 of 9 sites), whereas this is only the case for one of the nine sites in the edCas9 (1.1) samples. This result may be due to a combination of a shift in binding stoichiometry caused by the lack of a high-affinity site for the dCas9/gRNA complex to occupy and higher selectivity conferred by the edCas9 (1.1) mutations relative to the dCas9. This sequencing data has also highlighted a key consideration when using a catalytically inactive version of the Cas9 protein for experiments such as chromatin affinity purifications. The introduction of exogenous DNA such as plasmids should be limited to single copies if possible to decrease the chance of enrichment of non-target sequences that may complicate data analysis.

## Conclusions

The goal of this study was to better understand the global specificity of dCas9 in the context of chromatin binding in *S. cerevisiae*. Overall, we find binding of both versions of dCas9 in yeast to be highly specific, with virtually no consistent binding of any non-repetitive off-target sites within the genome. CRISPR technology has come a long way in just a few years. Because of the many technical advances to both the Cas9 enzyme and gRNA, the many methodologies surrounding this technology have been refined and are becoming more effective with less off-target interference. These refinements can have varying impacts depending on the CRISPR application, which is why it is necessary to evaluate them in each setting to fully understand their implications.

##  Supplemental Information

10.7717/peerj.9442/supp-1 Figure S1 *ARS305* locus with annotations

10.7717/peerj.9442/supp-2 Data S1 RTPCR raw data used for Fig. 2

10.7717/peerj.9442/supp-3 Data S2 Plasmid map of pPrA-dCas9 + gRNA-ARS305 ADH1 promoter plasmid The annotated plasmid sequence of the expression plasmid used in this study which includes the ADH1 promoter driven dCas9 gene.

10.7717/peerj.9442/supp-4 Data S3 pPrA-dCas9 + gRNA-ARS305 GAL1 promoter plasmid The annotated plasmid sequence of the expression plasmid used in this study which includes the GAL1 promoter driven dCas9 gene.

10.7717/peerj.9442/supp-5 Data S4 pPrA-edCas9 (1.1) + gRNA-ARS305 GAL1 promoter plasmid The annotated plasmid sequence of the expression plasmid used in this study which includes the GAL1 promoter driven edCas9 (1.1) gene.

## Additional Information and Declarations

### Data Availability

The following information was supplied regarding data availability: The ChAP-sequencing data are available at NCBI: PRJNA574983.
