# Supplementary material for: Genome-wide Cas9 binding specificity in Saccharomyces cerevisiae
Source: PeerJ. 2020 Jul 29;8:e9442. doi: 10.7717/peerj.9442 (PMC7395602; doi:10.7717/peerj.9442)
Supplement: Figure S1 [file peerj-08-9442-s001.pdf]

>ARS305 SGDID:S000028482, chrIII:39508..39595 1kb

GATTACAGGGAATTTGTTTAATAGCAATTTATACGCTTTGTTATCGGCACCACCAAATTCTGGGATAACCGTTAATT
CTTCCTCAGGTTTGCCTAGTGGATCCTCTCCTTCTGGAGTTTGGCCACGCTCTGGCTTTTCGATCAGACTTGGCATG
TGACTAATCAAGTATGGCATGCTGGTTTTTGGGTCCTTTGTTTTCGTTGTTTCAGTCTGGATAAATTTTAAGTTACCA
TTATCGAAGGCACTTTTGTACTTGTCACTAATTAAAGATGCAATGTCAGCGGGGATACTCATTTTTATTTTAATTTTT
ACTTTTCTGTTTGTTCTAAAATCTATCTAAACTGGCTTTCAAGATCAATCTATTGTCTTTTAAGGTAAACTTTAAATTG
GAAATAATAGTAATGTTAGTTCCTTCATTTTAACCTTGTATTGTATTTCCTTTGCGTGATGAAAAAAAAACTGAAAA
AGAGAAAAATAAGAAAATCTTCTAGAACGTTCCGAAACAGGACACTTAGCACACAAATACAGAATAGGAAAGTAA
AAGGCAATATATGAATGCAGTGCTTGTAACTGGTGCTTGTATCCAAGAATAGCTTCTTGCTGTAGGTTATGGGAAT
ATCGTGTAAGCTGGGGTGACTTTTGAGCTATTCGCGACGCCCGACGCCGTAATAACTACTTTCGACAGACCACTTA
TGACAGTATTTCAGGCCGCTCTTATAAAATGACATGTTAACAAACAGTTCTGATTATTCGCCTTTTGACAGGACGAT
AATGTAAATAGTTGTGGTAGTATCATTCAGGTATGTAACTGTTTACTTTGTATCGCTTGAAAAAAATAAGCATTTCA
GAGCCTTCTTTGGAGCTCAAGTGGATTGAGGCCACAGCAAGA*CCGGCCAGTTTGAATGCTCAACTCTTCAAAAGAA*
*ATTCCTCAAATATGT**CCAGTTTCATGTACTGTC**CGGTGTGATTTATTATTTTTTATTTACTTTGTAGTTCTTAAAGCTA*
AGATTTTTTTCTTTGATAAATTCTTGTTTTCATATCCTAAAATTAAAGGGAAAATAAACAATACATAACAAAACATAT
AAAAACCAACACAATAAAAAAAAGGATCAAATACTCATTAAAGTAACTTACACGGGGGCTAAAAACGGAGTTTGA
TGAATATTCACAAGATAAAAATCATATGTATGTTTCTGATATATCGATATACAATCAAACACTTTCAAGAATTTGTTT
*GTAGACTTTTTGCTAGAGACCT**CATCAAAGTGCTACCAAC**TAAGATCAACTTATACTTCTTTTAGAGAAAATTTTTTT*
*CAATGTACTCCAAAGAGATTTAGATCCTGTC*TCTTCCTCTTCCTCTTCCTCGAAAGTCAAAGAAAAATCAGAGTCTCC
CTGCTTATTCAGGCGGAGAGGCTCTAGGGTAGTTGCGTTTCTCTCATTGGGACACTGAACCTCATTTTCCAACATTT
TGGTCATGTAAGAGGCGACAGGCTCATCGCAGGTGGGTGCATCAACATGGTAGTACCTGGACCAAGCGCTACATT
GAGTCCCTCCTGGATAAACACCGCTACAATATTGTCTTTGGACGTTTGCCCAAACCATATCTTTTGAATACCAAAGC
TGGACCACATTGTATGGCCTAATCATTGGTGCTACCATAATACTGGATTGGGAAACAGTCTGGTTAATTTTTTTCAA
CCAATTTTTCTTATCTAGCAATGATTTAATAAACCTGAAATCTAAATTGTCTTCGTTAGCGTCTGTGTCATAATCTAC
AATTGAGTACTGTGACGTCCAATTATATGGCACCGAGATGGGGAATCTGTCCGGTGTTTCGTCGCTGTTATCCTTCT
CCTCCCTCCAAATGCAGTCAGAGGCAGGTGCCCATTCGGTTCGCCAGTCTCCGTTATTTACTACTTGGTACTGTTCC
CAATCGTAATACGTTTCCTCTGGGTTGAAGATACTTGCTCTGCTCTTGACATTGCCCATAGCCACACCACGAGAAAC
ATCGTGGAAGATTACGGAGCTGTTTACGATAGCAGGAGCAATGGATTTGACGAATGACACTTGATAAAAGTCTTT
GGTCGAAAA

Figure Legend:

ARS 305-1 gRNA target site: GTTGGTAGCACTTTGATG

ARS 305-2 gRNA target site: CCAGTTTCATGTACTGTC

Bold and Underlined: gRNA target sequences

Italics: Sequence deleted and replaced with URA3 cassette in control strain

Yellow highlight: PAM sequence

Green highlight: ARS305 sequence as listed by SGD (Saccharomyces Genome Database)

Red highlight: ARS305-2 qPCR primer set

Brown highlight: ARS305-1 qPCR primer set
